# A Review of Measurement Calibration and Interpretation for Seepage Monitoring by Optical Fiber Distributed Temperature Sensors

**DOI:** 10.3390/s20195696

**Published:** 2020-10-06

**Authors:** Yaser Ghafoori, Andrej Vidmar, Jaromír Říha, Andrej Kryžanowski

**Affiliations:** 1 Faculty of Civil and Geodetic Engineering, University of Ljubljana, Jamova cesta 2, 1000 Ljubljana, Slovenia; andrej.vidmar@fgg.uni-lj.si (A.V.); andrej.kryzanowski@fgg.uni-lj.si (A.K.); 2 Faculty of Civil Engineering, Brno University of Technology, Veveří 331/95, 602 00 Brno, Czech Republic; riha.j@fce.vutbr.cz

**Keywords:** optical fiber DTS, temperature, seepage, calibration, data interpretation

## Abstract

Seepage flow through embankment dams and their sub-base is a crucial safety concern that can initiate internal erosion of the structure. The thermometric method of seepage monitoring employs the study of heat transfer characteristics in the soils, as the temperature distribution in earth-filled structures can be influenced by the presence of seepage. Thus, continuous temperature measurements can allow detection of seepage flows. With the recent advances in optical fiber temperature sensor technology, accurate and fast temperature measurements, with relatively high spatial resolution, have been made possible using optical fiber distributed temperature sensors (DTSs). As with any sensor system, to obtain a precise temperature, the DTS measurements need to be calibrated. DTS systems automatically calibrate the measurements using an internal thermometer and reference section. Additionally, manual calibration techniques have been developed which are discussed in this paper. The temperature data do not provide any direct information about the seepage, and this requires further processing and analysis. Several methods have been developed to interpret the temperature data for the localization of the seepage and in some cases to estimate the seepage quantity. An efficient DTS application in seepage monitoring strongly depends on the following factors: installation approach, calibration technique, along with temperature data interpretation and post-processing. This paper reviews the different techniques for calibration of DTS measurements as well as the methods of interpretation of the temperature data.

## 1. Introduction

Seepage flow as a hydraulic load, when combined with erosion susceptible material and unfavorable stress condition in embankments, may initiate internal erosion [1]. This process is the cause of about half of all dam failures [2] and more than one-third of the accidents in levees [3]. Seepage development is a slow process, which requires continuous monitoring. Traditional methods (e.g., measuring pore pressure using piezometers and measuring the leaking water using weirs) are mostly unable to detect seepage in the initial stages due to punctual measurements in time and space [4], therefore, a seepage monitoring system with continuous measurement both in time and space is required.

Thermometric analysis is a seepage monitoring technique that was developed by Kappelmeyer, 1957 [5]. The method is based on the analysis of the heat transfer process in the soil. Heat is transferred in the soil through three mechanisms; conduction, convection by the percolated water, and sun radiation [6]. Since radiation duration is short and it affects only the surface layer of the soil [7], it can be neglected during thermometric analysis for seepage monitoring. Considering the heat transfer process, the thermometric method is defined as continuous or time-periodic repeating measurements of the temperature in the ground to trace the groundwater flow.

The heat transfer process in the soil can be described using the energy balance equation in porous media. Considering the local thermal equilibrium and neglecting the dispersion effects, the simple energy balance equation in the soil can be obtained by taking the average over a representative elementary volume (REV) of the soil [8]:

(1) $$\rho c\frac{\partial T}{\partial t}+\rho_{w}c_{w}.\;v.\nabla T=\nabla \cdot \left(\lambda \;\nabla T\right)+q^{\prime \prime \prime }$$

where ρ,c and λ are the soil density, specific heat capacity, and thermal conductivity respectively. T is the local temperature of the soil. v is the Darcy velocity of the flow. $\rho_{w}$ and $c_{w}$ are the water density and specific heat capacity and $q^{\prime \prime \prime }$ is the overall volumetric heat generation in soil. The heat capacity and the thermal conductivity of soil are related to the thermal properties of solid particles, fluid, and gas states in the soil.

Equation (1) presents the thermal energy transferred by the soil particles, the water in the pores, and the air trapped in the voids. The second and third terms of the equation present the heat transferring due to convection and conduction processes, respectively. The convection term ($\rho_{w}c_{w}.\;v.\nabla T)$ presents the direct relation of the heat transfer process with the seepage flow within the soil. Additionally, the presence of moisture affects both the thermal conductivity and the specific heat capacity of the soil. Therefore, both seepage flow and the presence of humidity in the soil influence the temperature distribution in the embankment [9].

By propagating the seepage into the embankment, the temperature of the water reservoir influences the thermal condition of the embankment. The convection by the seepage flow will dominate the heat transfer process even at a very low Darcy velocity in the order of $10^{-6}$ m/s [10]. In the seepage zone, the seasonal temperature variation of soil mostly depends on the temperature of inflow water, the seepage flow rate, and the distance from the inflow section to the measured point [6,7,11]. Rising the seepage velocity will increase the influence of the convection process. A significant difference is observed in the temperature of the embankment body between the zones with fast and low seepage velocity [9].

The thermometric method can be applied by measuring the natural temperature of the embankment body called the passive method. However, one can characterize the seepage by applying heat to the embankment and monitoring its dissipation, called the active method. Kappelmeyer [5] introduced the thermometric method by embedding a thermometer in a shallow soil layer. Later, the thermometric analysis was performed using the temperature measurement within the existing piezometers and standpipes in embankment dams. In 1991 another technique was developed for temperature monitoring using hollow pipes with several integrated temperature sensors [10], which provided more measurement points in a vertical profile. Using the thermometers for seepage monitoring allows for a continuous measurement in time, however, the monitoring system was still subjected to some shortages because it measured the temperature only in the vertical profile rather than cover a wide area.

Parallel to the advances in the thermometric method, the optical fiber technology was developed within a few decades. In the 1970s the distributed temperature sensor (DTS) based on Raman spectroscopy in optical fibers was invented [12,13]. The use of optical fiber DTS in thermometric analysis provides the opportunity to expand the areas of investigation and monitoring [2] with high sensitivity, lower cost, and smaller influences on the mechanical properties of embankment materials [14,15]. An understanding of the technical performance of the DTS and the system’s instrumentation is required for proper system employment and to improve the analysis of raw temperature data, which are acquired by the system measurement. To obtain a precise temperature, the DTS measurements need to be calibrated. However, the temperature data do not provide any direct information about the seepage. These data need to be processed and analysed to obtain the required seepage-related information. An efficient DTS application in seepage monitoring strongly depends on the following factors: installation approach, calibration technique, along with temperature data interpretation and post-processing.

To optimize the application of DTS in seepage monitoring, proper calibration and interpretation techniques should be selected. The objective of this paper is to review the present techniques and methods for the DTS data calibration and temperature data interpretation. This paper provides information about the potential advantages and disadvantages of various techniques, presents a comparative overview of them, and clarifies the possible topics for further studies. The current review provides helpful information for selecting the appropriate method for data calibration to obtain the precise temperature required in structural health monitoring systems as well as the selection of proper interpretation techniques for early detection of the seepage in embankments.

## 2. Raman Based Optical Fiber Distributed Temperature Sensor

A distributed temperature sensor (DTS) is an intrinsic fiber sensor system [16] which uses the Raman spectroscopy technique in an optical fiber to measure the temperature. When light photons with sufficiently high energy (e.g., visible light) interact with molecules of matter, a vibrational energy transition occurs [17]. During this process, the molecule absorbs and re-emits a portion of pulsed light with the different moving directions and with the same, lower or higher photon energy. Raman spectroscopy is a vibrational spectroscopic technique which is related to the movement of atoms in the glass of a fiber.

In the scattering process, most of the photons are scattered with the same energy level as that of incident light, with no energy transfer occurring [17,18] between the scattered light and the molecule. This scattering process is called elastic or Rayleigh scattering. As a result of the interaction process of the photon with the molecule, a very small portion of the light is scattering as Raman scattered photons, which is an inelastic process. Two types of Raman scattered photons can be described by the process of the vibrational energy level transition within the interaction event of the photon and the molecule; Stokes and anti-Stokes. If initially, the molecule is in the ground energy state, it is excited by the photon to a virtual state and then falls to an excited state. So in this process, the molecule promotes from a ground energy state to a higher excited vibrational energy state [18]. This process is called Stokes scattering. In Stokes scattering, the molecule absorbs the energy from the scattered photon and the photon is scattered with a smaller energy level. However, if the molecule is initially at an excited state, it rises to a virtual state by the photon and then falls to a ground energy state. This process, which involves the transfer of energy from the molecule to the scattered photon, is called anti-Stokes scattering. In both Stokes and anti-Stokes, the molecule and photon acquire different vibrational energy than that in the initial state. Since the energy of the photon is directly proportional to its frequency by the Planck’s constant [18], the scattered photons propagate with different frequencies and wavelengths than those for the incidence light.

The physical condition of the cable and the external factors such as strain and temperature affect the fiber molecules and consequently the scattering process. Different optical fiber sensors are developed based on the detection of the scattered photons to measure the temperature and the strain. While the Raman scattering is a temperature-dependent process, the Rayleigh and Brillouin scattering are employed in some optical fiber sensor systems to measure the strain [19,20]. The vibrational energy level of the molecules in the fiber cable strongly depends on the temperature that the cable is exposed to. At the ambient temperature, most molecules are presented in the ground energy vibrational level and the Stokes scattering forms the major part of the scattered photons [21]. The temperature raise excites the molecules and increases their vibration energy level. In the interaction of the photon with such a molecule, the photon absorbs the energy and is re-emitted as the anti-Stokes scattering. Therefore, the intensity of anti-Stokes scattered photons increases relative to the Stokes with raising of the temperature in the measured segment. The change in temperature of the fiber influences the intensity of backscattered light and the intensity ratio of Stokes and anti-Stokes backscattered light can be employed to monitor the spatial temperature field where the optical fiber is laid [18,21].

Long [22] stated that the ratio of anti-Stokes to Stokes intensities $R\left(T\right)$ depends on the temperature and the wavelengths of the scattered lights:

(2) $$R\left(T\right)={\left(\frac{\lambda_{s}}{\lambda_{as}}\right)}^{4}\exp\left(-\frac{hc_{l}\nu_{n}}{KT_{c}}\right)$$

where $\lambda_{s}$ and $\lambda_{as}$ are the wavelengths of Stokes and anti-Stokes, respectively, h is the Planck’s constant, $c_{l}$ stands for the light velocity, K is the Boltzmann constant, and $T_{c}$ represents the absolute temperature. $\nu_{n}$ is the wavenumber which represents the difference between wavelengths of incidence light $\lambda_{inc}$ and scattered lights $\lambda_{sc}\;$:

(3) $$\nu_{n}[{\mathrm{cm}}^{\;-\;1}]=\frac{10^{\;-\;7}}{\lambda_{inc}\left[\mathrm{nm}\right]}\;-\;\frac{10^{\;-\;7}}{\lambda_{sc}\left[\mathrm{nm}\right]}$$

**Figure 1:**
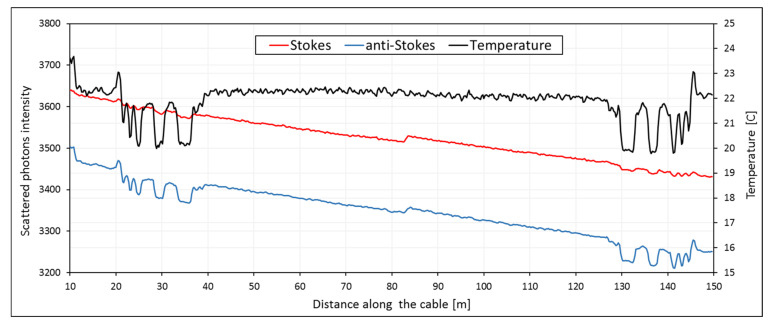
Stokes and anti-Stokes intensity and obtained temperature from the laboratory DTS measurement using the Silixa XT-DTS system.

Equation (2) is the basic equation for temperature measurement using Raman spectroscopy. This equation has been later developed and employed on the optical fiber systems for temperature measurement. Figure 1 shows the resulting temperature and Raman scattered intensities for a laboratory measurement. Duplexed single-ended measurement has been performed while several sections of the cable were replaced in cold water. The loss of the scattered light with the lengthening of the cable and the direct dependency of the anti-Stokes scattered photons on the temperature variation can be observed.

Optical fiber DTS employs the optical cable which consists of a transparent silica core, surrounded by the cladding and mechanically protected by a protection cover. The core and cladding both consist of silica (${\mathrm{SiO}}_{2}$), while the core is also doped with some other matter (e.g., ${\mathrm{GeO}}_{2}$) to raise its refractive index. The system uses the optical time-domain reflectometry (OTDR) technique, which was invented in 1989 [23] to measure the ambient temperature [24,25]. In this technique, the system launches short pulses of laser light into the fiber optic cable core to generate an infinitesimal amount of Raman backscatter lights, which are generated due to energy transfer between the light and the optical cable core molecules. The system measures the travel time of the light and captures the generated backscattered lights at regular time intervals by a fast photonic detector [26]. The intensity ratio of the Stokes relative to the anti-Stokes is measured and the absolute temperature of the sample is governed [18]. A great advantage of the OTDR technique is that the ratio of Stokes/anti-Stokes scattering is unaffected by changes in the geometry of the fiber, laser power, and cross-section of the detector or impulse pump [27].

In the optical fiber, the efficiency of the laser is proportional to its frequency [18] and its power decreases due to the attenuation process which consists of absorption, fluorescence, and scattering. The last one is used for temperature measurement by the system. The other two factors should be minimized for an efficient system. Absorption will occur in certain wavelength bands, therefore the system avoids this attenuation using the wavelengths other than the absorption band [28]. The fluorescence occurs when a very short wavelength is used, and the molecule reaches a new electronic energy level [17] rather than vibrational energy change. The system avoids fluorescence by the use of time-dependent measurements, using mathematical post-processing or recording two slightly different Raman spectra [28].

During the Raman scattering process, the Rayleigh scattering is the most intense part of the scattered light that can be harmful to the highly sensitive photodetector due to its high intensity. In addition to that, the Rayleigh scattering is irrelevant to the temperature measurement, therefore it is treated as system noise and should be filtered by the DTS. Additionally, to count the Stokes and anti-Stokes scattered lights and obtain their ratio, they should be separated [12]. The scattered lights can be filtered and separated based on their different wavelength and frequencies.

The change in the temperature of the fiber will affect the intensity of backscattered light and the intensity of Raman scattered photons will be modulated to determine the spatial temperature field where the optical fiber is laid [21]. In dam safety monitoring, the local temperature changes may occur due to the convection heat transfer by seepage propagation. The temperature change affects the optical properties of the fiber and consequently the optical signal frequency characteristics.

**Figure 2:**
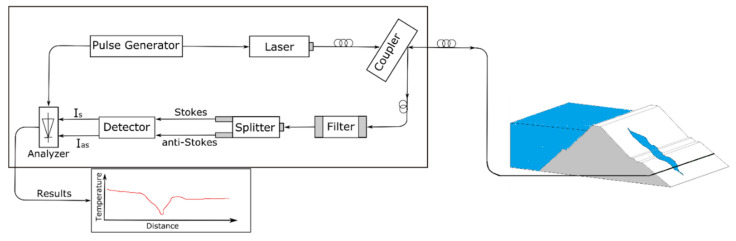
The basic components of an optical fiber DTS system. Adapted from [30]. (The publisher gave permission to reproduce this figure.).

The optical fiber DTS systems generally consist of some basic components [21,25]. The light pulses are generated by the pulse generator and the laser launches the generated pulse of light into the optical cable. The intensity of the laser strongly influences the precision of the measured temperature. The system guides the launched lights into the cable core using a coupler, which is also employed to guide the backscatter photons into the detector system. The launched lights are traveling into the optical fiber cable, which acts as a continuous, distributed sensor for temperature. DTS system is equipped with a powerful optical filter to allow the Raman signal and prevent the intense Rayleigh signal. The other crucial component of the system is the detector that detects the backscattered Raman lights. Since the Raman effect is weak [28], a sensitive detector is required in optical fiber DTS systems. In addition to the laser intensity, the precision of the measurement strongly depends on the detector sensitivity [29]. The optical fiber DTS is also equipped with an optical spectrum analyser system that resolves different wavelength peaks (Stokes and anti-Stokes) and the corresponding software that calculates the temperature difference and the location from which the backscattered light is detected. The optical fiber instrumentation is demonstrated in Figure 2.

The optical fiber DTS system can be installed in three different configurations, the so-called simple single-ended, duplexed single-ended, and double-ended configurations. In a simple single-ended configuration the cable has one connection to the instrument and the system measures one temperature in a point along the cable length from the DTS outward. In the duplexed single-end approach, the cable is installed to DTS in one connection, but it consists of two co-located fibers that are following the same path. This configuration allows the system to report two temperature observations in every point of the cable. One from the DTS outward and the other from the cable coming back to the instrument. In the double-ended configuration, the cable is connected to the system from both ends and allows the system to observe the temperature from both directions [31].

**Figure 3:**
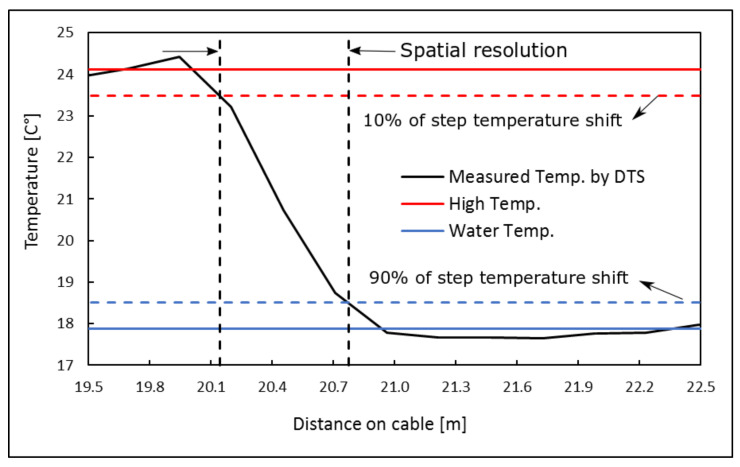
The spatial resolution of the Silixa XT-DTS system.

To interpret the measured temperature by the DTS system, understanding of the systems’ parameters such as spatial sampling, spatial resolution, temporal resolution, accuracy, and power losses are crucial. These parameters are related to each other and significantly influence the final temperature measurement. The system sampling resolution is the shortest distance between successive temperature measurements. However, the reported temperature in each sampling interval is not independent of its adjacent reported temperatures [32] and a step-wise temperature shift may not be measured accurately by one sampling interval length. The spatial resolution is then defined as the distance between two points reporting 10 to 90 percent of the true temperature of a step-wise shift in temperature along the optical fiber cable [33,34]. The spatial resolution is usually larger than the sampling interval. We examined the spatial resolution of the Silixa XT-DTS system with a 25.4 cm sampling interval, 150 m of the measured length, and an integration time of 10 s. A temperature step shift was modeled by immersing 3 m of the optical fiber cable into a water bath with a constant temperature of 17.9 °C. The result shows a spatial resolution that equals 0.63 m (2.5 times larger than the employed sampling interval) for the system, as can be seen in Figure 3.

The other crucial parameter in the employment of the optical fiber DTS is the integration time of the system. This is the time that the system requires to report one temperature profile over its entire length [15]. Most DTS systems allow us to adjust the integrated time before the measurement. A longer integration time gives the system enough time to detect a larger amount of backscatter photons and increase the measurement precision in the long-distance measurement.

## 3. Calibration of Optical Fiber DTS Temperature Data

**Figure 4:**
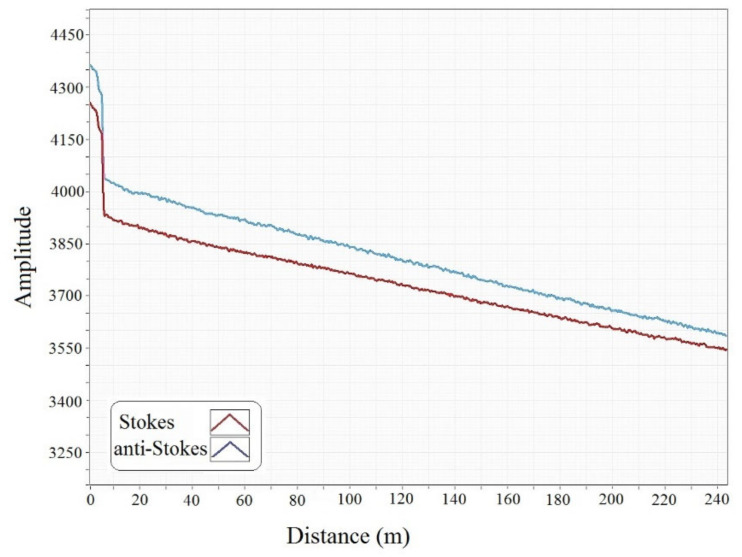
The loss in the intensity of Stokes and anti-Stokes scattering due to the connection of two types of optical fiber cable.

Attenuation in the optical fiber significantly influences the temperature results in the system. In addition to the mechanical attenuation by the system and the physical attenuation in the cable (such as connectors, splices, and bends), the strength of the optical signal decays exponentially with distance from the source (Beer’s law) [29,35]. The longitudinal attenuation in the multimode fiber which is used in DTS, is relatively higher than the single-mode fiber. This attenuation sometimes limits the range of the distance for Raman-based DTS measurement to approximately 10 km [36]. Figure 4 demonstrates the power losses of the system in terms of decays in the intensity of Raman scattered photons due to the length of the cable and the presence of a connection at the distance of around 7 m from the system. A fusion splicer was used to connect an OM2 fiber cable to the OM3 cable patch. Both OM2 and OM3 have cores with a diameter of 50 µm, while OM3 is optimized for laser-based equipment that uses fewer modes.

DTS systems consider attenuations and provide an internal calibration for temperature measurement. In addition to the system internal calibration, manual calibration is required for many practical applications. The objective of both internal and manual calibration is to eliminate the effect of attenuation to obtain accurate temperature measurements. To optimize the DTS employment for temperature measurement in seepage detection, the selection of an efficient and reliable calibration approach is essential.

Commonly, the DTS system corrects the longitudinal attenuation by introducing a linear power loss per length of the fiber with the unit of dB/length. Also, the DTS system internally calibrates the temperature measurements by assigning a reference section or reference point of the cable where the temperature on the cable is known [34,37] and monitored continuously by the precise thermometers which are attached to the system. In practice, the reference temperature can be determined by immersing part of the optical cable in a water bath at a stable absolute temperature. The DTS instruments such as power suppliers, laser, and detectors are temperature sensitive and may interfere with the temperature measurement process in the system. DTS systems usually calibrate this sensitivity and eliminate its effect by a reference coil of fiber [31] which is commonly replaced between the directional coupler and the sensing fiber [38]. The system continuously measures the temperature of the reference coil with a precise internal thermometer and using scatter analysing and compares them to correct the instrumental errors.

In addition to the temperature data, which result from internal instrumentation calibration, the DTS system also provides raw data which are the obtained intensity of Stokes and anti-Stokes backscattered photons. Manual calibration techniques use these raw data and consider different interference factors to obtain more accurate temperature results. In other words, the manual calibration partially eliminates the role of the analyser unit from the DTS and calculates the temperature results from the detected Raman scattered photons.

Farahani and Gogolla [39] introduced an equation to calculate the temperature from the obtained Raman intensity data, which was used later as the base of manual calibration techniques. The equation extracts the temperature at a distance *z* [m] along the cable from the detected power of Stokes, $P_{s\left(z\right)}$, and the power of anti-Stokes $P_{as\left(z\right)}$:

(4) $$T\left(z\right)=\frac{\gamma }{ln\frac{P_{s\left(z\right)}}{P_{as\left(z\right)}}+C\;-\;\Delta \alpha z}$$

where $T\left(z\right)$ is the temperature [K], γ [K] is related to the energy shift between the incident photon and the Raman scattered photon, *C* is a dimensionless calibration parameter which represents the influences of the incident laser properties and the DTS instrument itself, and Δα [$m^{\;-\;1}$] is the differential attenuation between the anti-Stokes and Stokes signals [40]. It should be mentioned that these three parameters are independent of each other. The manual calibration is based on the techniques for seeking the three calibration parameters γ, *C*, and Δα using reported values of Stokes and anti-Stokes power and independent temperature measurements at reference locations.

Four calibration approaches can be used to obtain the calibration parameters for a single-ended installation configuration [31]. In the first and second algorithms, the independent calibrating parameters can be obtained in the presence of three reference points (first approach) or three reference sections (second approach) with known temperatures. Equation (4) can be solved for the calibration parameters with these three known temperatures and known intensities of scattering. The three calibrating parameters can be found by simultaneously solving a set of linear equations.

The third and fourth approaches can be used for cases where three measuring references are not available. In these approaches, the value of Δα is obtained independently from the other two parameters. In the third approach, a long reference section can be used to calculate the value of Δα. Since Δα is the differential attenuation between the anti-Stokes and Stokes signals, the Beers’ law can be employed to determine the value of Δα using the reported Stokes and anti-Stokes power at two points ($z_{1}$ and $z_{2}$) of a cable section with a uniform temperature (see Equation (5)). The accuracy of this method is related to the uniformity of temperature along the reference section, the linearity of the Raman spectra data, and the length of the reference section or the number of point measurements considered in the regression:

(5) $$\ln\frac{P_{s\left(z_{2}\right)}}{P_{as\left(z_{2}\right)}}=\ln\frac{P_{s\left(z_{1}\right)}}{P_{as\left(z_{1}\right)}}+\Delta \alpha \;\left(z_{2}\;-\;z_{1}\right)$$

In the absence of an extensive reference section, the values of Δα can be calculated from two separate points ($z_{1}$ and $z_{2}$ from the DTS) at the same temperature, Equation (6) (fourth approach). The accuracy of this approach depends on the distance between two points. The longer the distance, the better accuracy:

(6) $$\Delta \alpha =\;\frac{\ln\frac{P_{s\left(z_{1}\right)}}{P_{as\left(z_{1}\right)}}\;-\;\ln\frac{P_{s\left(z_{2}\right)}}{P_{as\left(z_{2}\right)}}\;}{z_{1}\;-\;z_{2}}$$

**Figure 5:**
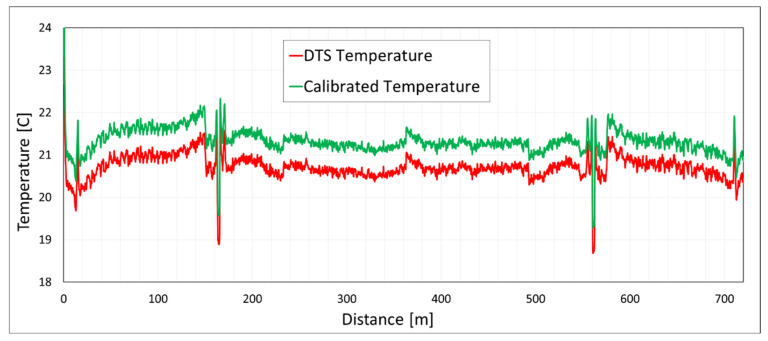
Temperature measurement with a duplexed single-ended approach and calibration based on two reference points for each side (four reference points in total).

Obtaining the value of Δα, the two other parameters can be found using the explicit calculation from the two reference points or sections. Knowing the three calibration parameters, Equation (4) can be employed to calibrate the temperature measurement along the entire cable. Figure 5 presents the temperature measurement by the duplexed single-ended installation configuration. 

The temperature results for both internal system calibration and manual calibration are demonstrated. The calibration was performed by having two reference points (four measuring points in both directions) and using the least-squares method for solving Equation (4).

The single-ended configuration assumes that a linear differential power loss and therefore a constant value of Δα exist along the entire cable. To estimate this value, considering a reference section of the fiber with a known temperature is essential at the end of the cable. However, in some field applications, providing a reference section at the end of the cable is not applicable. Also, in some cases, the assumption of a linear differential loss along the entire length of a cable is not valid, due to the presence of step changes in Δα along the cable associated with bends, connectors, splicers, and other irregularities. One can overcome these shortages using a duplexed single-ended configuration, where the system reports two temperature observations in every point of the cable. However, a duplexed single-ended configuration requires a two times longer cable than the single-ended to cover the temperature monitoring for the same investigation area. Hausner and Kobs [41] suggested that the step losses in the single-ended installation should be identified and corrected carefully by the calibration. They presented also a correction algorithm that best suits the duplexed single-ended installation.

In the double-ended configuration, where the system launches the laser into the fiber from each end, a unique value of differential power loss Δα can be calculated for each data acquisition section [42]. The differential losses along the entire cable length can be calculated by integrating Δα along all measuring sections. Using the double-ended configuration, one can locate the step attenuation as well as calculate the differential attenuation rate throughout the length of the fiber [34]. The double-ended configuration is an appropriate technique where the presence of step power losses along the optical fiber is a critical factor that can significantly influence the temperature measurements. Also, this technique can be used where it is not practical to place a reference bath at the end of the cable [43]. The double-ended configuration also can be used by the system to internally calibrate the measurements using the two reported temperatures for each measurement point.

The main shortage of the double-ended approach is its higher noise than the single-ended measurement [34] because it includes data collection from two different measurements with their own intrinsic noises from each end of the cable. In the double-ended configuration, a point located near the DTS is far from the system, when the measurement takes place from the other end of the fiber. This will cause noise in the calculation of differential losses especially at both ends of the cable [42].

**Figure 6:**
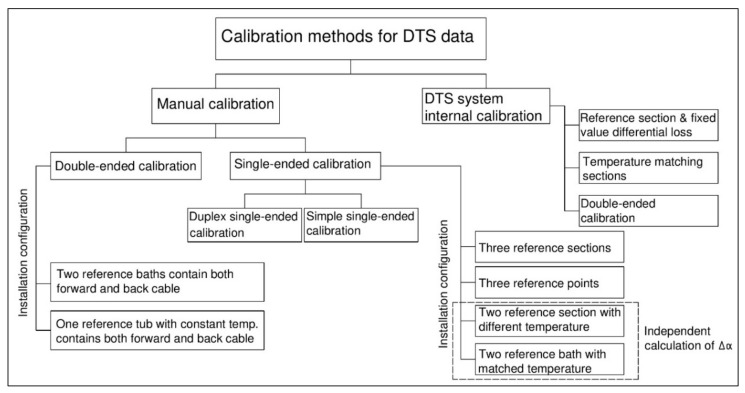
General typology of DTS data calibration.

Generally, the DTS data calibration can be classified as shown in Figure 6. Additionally, a comparative overview of the manual calibration techniques is presented in Table 1.

The DTS calibration might be employed statically or as a dynamic calibration. In the static calibration, the calibration parameters are obtained from the data taken over an initial integrated period. These parameters are assumed to be uniform along the cable and constant over time [38]. Due to the temporal variation of parameters, the static calibration is not valid for many applications and the dynamic calibration is required. The dynamic calibration recalculates the three calibration parameters for each measurement time step. The DTS system is capable to perform a fully dynamic calibration by defining the reference sections with constant temperature or integrated temperature probes [34].

The DTS systems are usually assessed based on their spatial resolution, integrated measuring time, absolute temperature accuracy, and the range of the optic cable that can be used for temperature measurement. This range presents the maximum length of the optical cable that the system can perform the temperature measurements without losing significant accuracy. Another important factor in the calibration process is the length of the reference section that should be embedded in the reference bath. This length should be usually longer than the spatial resolution of the DTS System. A reference section at least ten times greater than the spatial resolution is recommended [34].

## 4. Seepage Detection Techniques from the Temperature Measurements

The very first applications of the optical fiber DTS in thermal analysis of embankment dams were linked with the installation of the optical fiber into the existed piezometer standpipes and observation wells. These standpipes and wells are usually drilled to monitor the water level; however, they can be used also for temperature measurements. Optical fiber can provide a vertical profile of temperature in the dam bodies along the entire height of the standpipes. Later, some other installation techniques were developed in various dams. Construction of new dams, repair of the existing structures, dam height raising, and upgrading works in dams and embankments provide opportunities for engineers to employ the optical fiber DTS with new techniques and approaches. While for the new dams, the optical fiber can be installed within the dam body, it can be also installed in the crest during the dam raising, within the downstream toe [46,47], and within the different section of the dam during extension or upgrading. Installation of the optical fiber along the dam downstream toe is one of the most efficient techniques because most of the leakage paths come through this zone and it is very applicable, cheap, and easy to install the fiber in this zone [9].

**Figure 7:**
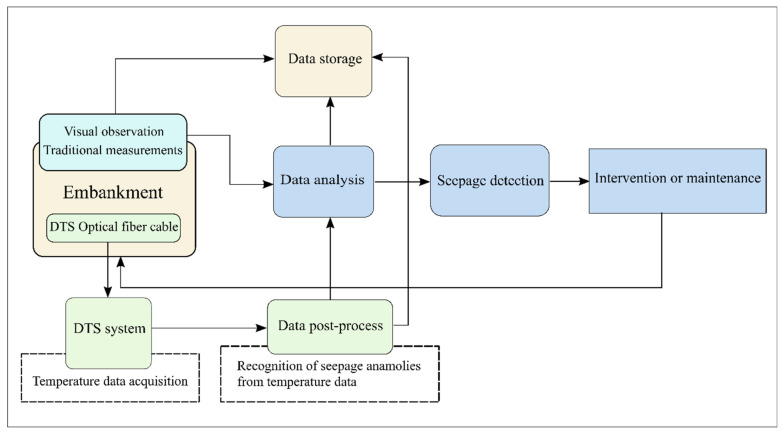
Monitoring and detection of seepage in the embankment dam.

Most methods for thermal analysis of seepage with the optical fiber require long-term measurements to provide enough information for proper interpretation of temperature data. Optical fiber DTS is employed as an indirect technique for seepage detection in embankments and earth-fill dams. DTS provides temperature measurements, which are not directly interpretable in terms of seepage and require further processing and data analysis to obtain the appropriate information about the seepage flow in the embankment. In addition to different approaches to the application and installation of DTS, the researchers introduced several techniques for analysing and interpreting temperature data that lead to seepage detection. The process of seepage monitoring in the embankments can be described as we show in Figure 7, which is a specialized form of the structural health monitoring (SHM) flow chart [48].

In this section, we reviewed the various methods for the installation of the optical fiber cable and techniques, which were developed for proper interpretation of temperature measurements.

### 4.1. Passive Method

Using the passive measurement method, the DTS easily provides large number of measurements of the natural temperature of the ground without any necessity to the power supply for heating. Passive DTS is used for long-term temperature monitoring in embankments and earth-fill dams. Some techniques were developed for the interpretation of acquired data from the long-term passive measurements.

#### 4.1.1. Lag-Time Method

The most common technique is based on the comparison of the temperature variation in the embankment body with the seasonal variation of temperature of the air and the reservoir water. Since the temperature in the embankment mainly depends on the air and water temperature at the reservoir, this technique can be employed for thermal analysis of seepage flow in the embankment. This technique assumes that the 24-hour temperature variation of air is negligible due to its short day-night cycle duration. The seasonal variation in air temperature and reservoir water creates the seasonal thermal response in the dam body [49]. This response depends on the seasonal air temperature, water temperature in the reservoir, and the distance from the reservoir to the measuring point [46]. The method requires precise monitoring of the water temperature in the reservoir of the dam. A lower temperature variation during long-term monitoring excludes the presence of a significant seepage path, while a larger seasonal variation may be a sign of seepage within the embankment [46]. The influence of air temperature is considerable only for the depth of a few meters [50]. Even the seasonal variation of air temperature affects the temperature of the embankment dam for the depths shallower than 10–15 m [7,10,51]. Due to the low thermal diffusivity of embankment materials, the heat pulse response for a measuring point at the depth of around 10 m may be as long as 6 months [10]. Long-term measurements of the water and air temperature, and the temperature within the embankment also can be used to estimate the flow velocity due to seasonal variation [10]. The lag-time method is one of the first methods for estimating seepage velocity. Johansson 1997 [7] applied this method to estimate the seepage flow from the long-term temperature measurements in the embankment. This technique is a simplified one-dimensional approach based on thermal velocity due to the convection process. The lag-time $t_{d}$ is the time between the temperature pulse of water and the air at the boundary x=0 and the temperature variation due to the boundary thermal pulse at the measured point at distance x [52]. The temperature may be considered as a tracer for the seepage monitoring that travels with a thermal velocity and not the pore water velocity. Assuming a one-dimensional thermal process, the thermal velocity ($v_{T})$ can be obtained from lag-time ($t_{d}$) and the distance of the measured point from the boundary (x) [52]:

(7) $$v_{T}=\frac{x}{t_{d}}$$

Considering the one-dimensional heat transfer by the seepage, the relationship between thermal velocity ($v_{T})$ and Darcy velocity (v) of the flow depends on the ratio of the soil and water specific heat capacities [53]:

(8) $$v=\frac{C}{C_{w}}v_{T}$$

It is very important to note that the lag-time method estimates the seepage velocity based on the domination of heat transfer due to advection. This means that the effect of conduction should be assumed negligible. This assumption is valid for a relatively large seepage flow and for the thick seepage zone where the vertical heat exchange due to conduction is negligible [7]. The advection process is the dominant heat transfer process for seepage velocity as low as 10^−7^ m/s to 10^−6^ m/s [10]. Methods based on the seasonal variation usually require long-term monitoring (in order of months and years), while some of the seepage associated erosion may develop very fast in the embankment. In addition to the lag-time method, Johansson [7] introduced the amplitude method for seepage detection using temperature measurements. The method assumes that the temperature varies sinusoidally at the upstream face, the advection occurs in one dimension, conduction is only vertical, and thermal properties are constant for each layer.

#### 4.1.2. Source Separation Method

Sometimes, in long-term measurements, detection of short-time leakage from the extensive data is not possible. There are many factors other than seepage that influence the heat transfer in embankments. These factors can be categorized as the ground response (e.g., permeability and physical composition), natural phenomena (e.g., seasonal temperature and rainfall), leakage, and existing drain structures [54]. The source separation technique is a statistical and signal processing-based analysis that had been introduced to process the thermal data and eliminate the irrelevant factors from the leakage information. This method is a medium-term monitoring technique that can be used to monitor leakage based on the months’ raw data [55]. The method is useful when a large number of acquisitions are provided by monitoring systems [56] as in the case of optical fiber DTS measurement. The known factors such as daily and seasonal effects and rainfall periods can be filtered from the data using the data filtering techniques such as low pass filter and Kurtosis based filtering [57]. This method considers the precipitation as an ephemeral phenomenon in the time domain and the leakage as an ephemeral phenomenon in the time/space domains [54].

At first, the raw data need to be arranged in a matrix (here, matrix Y) which contains the observed temperature data as a function of time and distance along cable $T\left(x,t\right)$. The method assumes that the different phenomena that affect thermal behavior are independent of each other. Considering this assumption, the data matrix can be formulated as:

(9) $$Y^{T}=MF^{T}$$

here, $Y\in R^{N_{x}\times N_{t}}$ is the data matrix as a linear mixture of independent sources, $M\in R^{N_{t}\times p}$ is the mixing matrix, and $F\in R^{N_{x}\times p}$ is the matrix of sources. p is the number of independent sources that affect thermal behavior in the embankment [54,56,57]. Equation (9) presents a source separation problem. As a pre-processing step, the data need to be normalized with zero mean [54]. Then the source separation technique employs different statistical techniques such as singular value decomposition (SVD) and independent components Analysis (ICA) to separate the leakage information from the entire temperature data. Using SVD, the matrix of data can be decomposed into two subspaces of $Y_{ground}$, which contains the nonsingular data and $Y_{useful}$ which contain the leakage information (Equation (10)). Then the useful data should be decomposed into subspace $Y_{leakage}$ and $Y_{rest}\;$(Equation (11)). SVD is not enough for separation of leakage anomalies from subspace $Y_{useful},$ therefore, the more realistic approach based on the ICA is applied to this subspace. ICA estimates the sources and their contributions to the mixture by considering mutual independence between the sources. Subspace $Y_{leakage}$ presents the temperature variation due to leakage and the associated time and location of those variations The SVD and ICA mathematical processes are presented in Equation (10) and Equation (11) respectively [56]. More details on the SVD and ICA and their applications in the source separation method can be found in [56,57,58]:

(10) $$Y=U_{N}\Lambda_{N}V_{N}^{T}=\sum_{j=1}^{m}\lambda_{j}u_{j}v_{j}^{T}+\sum_{j=m+1}^{N}\lambda_{j}u_{j}v_{j}^{T}=Y_{ground}+Y_{useful}$$

(11) $$Y_{useful}=\sum_{j=m+1}^{m+q}\lambda_{j}u_{j}b_{j}^{T}+\sum_{j=m+q+1}^{m+p}\lambda_{j}u_{j}b_{j}^{T}=Y_{rest}+Y_{leakage}$$

Application of SVD and ICA on the acquired data requires important decisions during the data processing. To apply the SVD and ICA, three parameters of *p*, *m*, and *q* should be assigned. *p* is the number of independent sources that should be decided, *m* is the number of singular values for building ground subspace, and *q* is the number of ICA sources to be estimated [54]. The selection of different values for these parameters may significantly affect the results.

The source separation technique was validated in the site measurement where the optical fiber was buried in the downstream toe of an embankment [57,59,60]. The measurement was performed for a certain time when the one artificial leakage and two drains existed in the embankment. The anomalies related to the drains and leakage were obtained by the application of one SVD and two ICA decompositions.

#### 4.1.3. Singularity Detection Method

The other statistical method for derivation of leakage information from the temperature measurement is the singularity detection method. In temperature measurement, most of the measuring points show a common trend of temperature variation in a certain acquisition time. However, the presence of singularities influences the trend of temperature variation. In the embankments, the possible singularities are due to the presence of leakages, drains, and the singularity of the ground response due to the different effects such as material heterogeneities [55]. These singularities present a deviation from the common trend of temperature variation related to the nonsingular distances. The method of singularity detection seeks dissimilarities in the temperature trend to determine the measuring distances associated with the singularity zones. Analysing daily measurements, this method can be used to create an alarm monitoring system for early leakage detection in embankments. If daily data analysis is performed, a resolution in a time of 24 h is obtained, which allows detection of anomalies as early as the second day of their development [59]. This method is based on the relative temperature variation of measuring points, therefore finding the precise absolute temperature is not a necessity.

**Figure 8:**
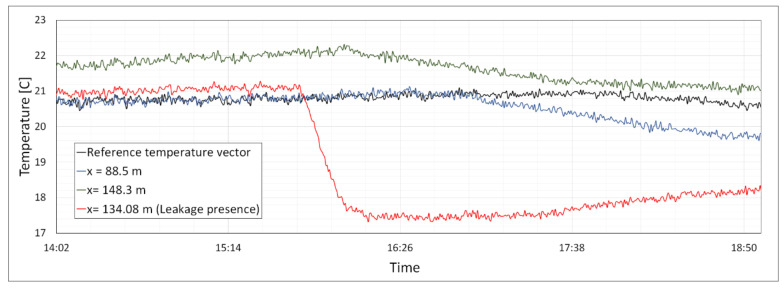
Singularity detection using short-term experimental measurement: Measuring point at x = 134.08 m corresponding to the location of a leakage path is the singularity that shows a different trend of temperature variation compared with the non-singularity zones.

Figure 8 presents the results of the singularity detection method using the temperature variation trend. A short-term experimental study was performed. The temperature trend for measuring point at x = 134.08 differs from the reference temperature trend. The increase in the deviation of the temperature variation trend for singular zones might be attributed to the increase in the flow rate of leakage.

The important step in the application of the singularity detection method is the estimation of a reference vector from the daily temperature variations, which can be compared with vectors at all measuring points [55]. The SVD technique can be used as a proper approach to determining the reference vector in the singularity detection method. The data matrix can be decomposed into the two subspaces. While the first subspace contains the most energetic singular values that present the dominant temperature behaviour of the soil, the second subspace resulting from SVD contains the information associated with the vectors deviated from the reference vector [55]. The first subspace can be used as a reference vector.

#### 4.1.4. IRFTA Method

The next method, which allows for analysing optical fiber temperature measurements in dikes and embankments, is the Impulse Response Function Thermal Analysis (IRFTA). This method was developed for thermal analysis of seepage, especially from the temperature measurements at the downstream toe of dams [61]. This method is a statistical-based method that requires temperature measurements of at least two months of thermal monitoring [18]. IRFTA employs Green’s function for the coupled water and heat transfer in the soil [62]. The impulse response function, $h\left(t\right)$ can be written in the form of two parameters: [9,63]:

(12) $$h\left(t\right)\approx R\left(\alpha ,\eta \right)$$

where α is the signal damping factor and η is the time-lag that quantifies the time elapsing between the loading onset and its response by the system in the measuring point. This time-lag can be presented as days for long-term monitoring in an embankment. The impulse response function presents how the parameters of the input signal (here α and η) are modified by the thermal behavior of the dam. In other words, α presents the significance of the influence of the parameter on temperature measurements, and η presents how fast this influence occurs.

The thermal analysis of the downstream toe of an embankment is influenced by the air temperature at the downstream face and the water temperature in the reservoir. Therefore, the IRTFA function can be finalized in a four-parameter function in the following form [61]:

(13) $$T\;\left(x,t\right)=\theta_{c}+R_{w}\left(x,t\right)\times \theta_{w}\left(x\right)+R_{air}\left(x,t\right)\times \theta_{air}\left(x\right)$$

where $\theta_{c}$ is constant (e.g., initial temperature), $\theta_{w}$ and $\theta_{air}$ are the coefficients of the temperature loading of water and air, respectively, acting on the dam surface, and $R_{w}$ and $R_{air}$ are the impulse response functions for the water and air temperature loading, respectively. The impulse response function of water ($R_{w}\;$) is associated with the two parameters of $\alpha_{w}$ and $\eta_{w}$, the damping factor, and the time-lags of the water temperature response. $R_{air}$ is the response parameter related to the air temperature; $\alpha_{air}$ and $\eta_{air}$. If the optical cable is located in the dry zone the equation can be reduced by considering $R_{w}\left(x,t\right)=0$; when the cable is in the saturated zone then the effect of air temperature can be assumed negligible ($R_{air}\left(x,t\right)\approx 0$).

**Figure 9:**
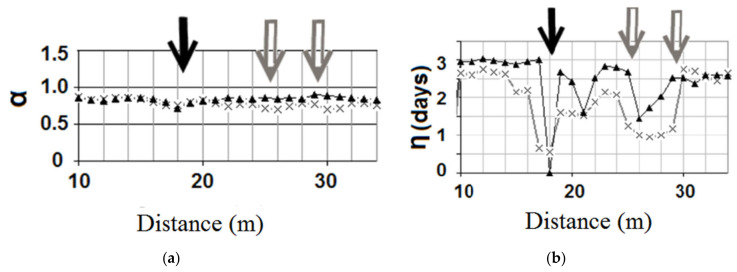
IRFTA analysis for the optical fiber temperature measurements in the dam downstream toe with three artificial leakage paths which are localized by arrows [9]: (**a**) The signal damping factor; (**b**) The time-lag. The cable is embedded in two different elevations at the downstream toe. The thicker line with triangle marks is associated with the temperature measurement by the cable embedded in the middle height of the dam toe while the other cable is embedded into the bottom level of the toe. (Figure reproduced with permission of the publisher).

The method was validated using a thermal analysis of seepage in a 27-m high dam [61] and a large model of a dam, while the artificial leakage was created by placing high permeable sand in the dam body [63]. The optical fiber was used to monitor the temperature variation in different levels at the downstream toe of the dam. Figure 9 presents the results of the IRFTA for the response function of water temperature ($\alpha_{w}$ and $\eta_{w}$) in a dam model with three artificial leakages.

The value of $\alpha_{w}$ is mostly influenced by the direct contact between the leakage water and the optical cable. This value is almost stable for any zone out of direct seepage velocity influences [9]. In Figure 9 this value is almost stable, therefore, it does not provide any information about the leakage presence. However, the leakages can be seen clearly in the $\eta_{w}$ graphs where the time-lag between the water temperature and the measured temperature in leakage zones is significantly dropped. The figure shows that the fiber buried below the seepage path detects the leakage more efficiently.

IRFTA is not only a statistical model as its parameters also provide a physical interpretation [9]. In addition to leakage detection, the IRFTA method provides information about the parameters that influence the temperature variation, quantifies the significance of these parameters, and estimates how fast these parameters influence the temperature distribution. However, the model describes only the linear behaviour of heat transfer, and in the case of high nonlinearity in the heat transfer process, the method may not be able to model the temperature measurement perfectly [61].

### 4.2. Active Method

Parallel to the passive DTS measurement, the active DTS application has been developed for many years. The active thermal analysis of dams and embankments for seepage detection was first applied by Dornstädter in 1997 [10] by introducing the heat pulse method. He installed a linear heat source into the dam body and studied the heat dispersion. Since the seepage flow increases the heat loss of the heating cable, the loss of the heat can be used as a tracer of seepage velocity. In the case of no seepage flow, the heat dissipation and cooling process are slow while the cooling process in the zones with a high flow is much faster. The duration of each heat pulse varied between 6 to 12 h. The active method was developed for the applications where there is no adequate seasonal temperature variation of the water reservoir or if there is not enough temperature gradient between the embankment body and the water [64]. This technique provided some advantages since it does not require the long-term seasonal temperature variation and provides a temperature gradient through heating the medium. Perzlmaier et al. [65] developed the active thermometry technique to be used with the optical fiber DTS measurements. This method is based on introducing heat along the optical cable for a few hours with A.C. or D.C. voltage. They estimated the seepage velocity as well as the degree of saturation in the vicinity of the fiber cable using the measured heat dissipation along the cable.

The heating along the cable can be applied in two approaches. In the first approach, a metal wire is embedded in the embankment parallel to the optical cable [14,65]. The linear ohmic resistance of the metal wire will produce linear heat from the introduced A.C or D.C. voltages. In the second approach, the heating wire is replaced with the optical cable in the same coating, which is called the hybrid cable [51]. Some of the fiber optic cables are composed of optical fibers enclosed and protected by the stainless-steel tubes or metal wire. The active method in these cables can also be performed using the metallic components as electric resistance heaters [66].

To estimate the seepage velocity with a high accuracy, Su and Kang [14] introduced an inversion technique based on the active optical fiber temperature measurements. They used the heat transfer equation (Equation (1)) which is simplified for 2D steady-state ($\frac{\partial T}{\partial t}=0$) heat transfer, constant seepage velocity (v), and only in the x-direction:

(14) $$\frac{\partial^{2}T}{\partial x^{2}}+\frac{\partial^{2}T}{\partial y^{2}}\;-\;\frac{C_{w}\rho_{w}v}{\lambda }\frac{\partial T}{\partial x}=0$$

Two optical fiber cables were embedded in the medium for temperature measurement. Cable 1 was an armoured optical cable that was heated through the metallic component of the cable. Optical cable number 2 was embedded parallel to cable 1 with a certain distance to sense the temperature rise due to the heated cable. $T_{1}$ is the equilibrium temperature of optical cable 1 after being heated. $T_{2}$ is the measured temperature by cable 2 for a certain measuring point in the temperature field generated by the heated cable 1. While the initial temperatures in both cables and the initial temperature of ambient $T_{0}$ are known the heating will be applied. Using the inversion method, seepage velocity v is assumed. Having assumed the seepage velocity, Equation (14) is solved for the temperature to reach a proper approach to the measured temperature $T_{2}$ by the DTS. The procedure continues until an acceptable approach to the measured temperature $T_{2\;}$ is obtained. The study showed that more precise seepage velocity can be achieved by a higher heating power.

When the seepage starts to flow, the heat transfer between the heated cable and the water at the vicinity of the cable (Q) simply equals the convection heat transfer $Q_{v}$. The amount of the heat transferred from the heated cable to the water can be obtained by:

(15) $$Q_{v}=A_{a}h\left(T_{cable}\;-\;T_{water}\right)=Q$$

(16) $$h=f\left(v,l,\rho_{w},C_{w},\mu ,\lambda_{w}\right)$$

where $A_{a}$ is the area of heat transfer between the cable and water, h is the heat transfer coefficient, $T_{cable}$ and $T_{water}$ are the temperature of the heated cable and water, respectively. The heat transfer coefficient is a function of flow velocity v, structural size l, water density $\rho_{w}$, heat capacity $C_{w}$, dynamic viscosity μ, and thermal conductivity $\lambda_{w}$.

Using the equation of heat convection between the seepage water and the heated cable (Equation (15)), Su et al. [67] studied the seepage velocity by the optical fiber active temperature measurements in a laboratory model for the soil-concrete contact. If the water properties and structure size in Equation (16) are considered constant, then the heat transfer coefficient is only a function of flow velocity. They used the correlation between the characteristic dimensionless numbers like Reynolds number, Planck number, and Nusselt number to develop an equation for the heat transfer as a function of seepage flow velocity. Heating was introduced to the model by applying a stable voltage on steel bars that were installed adjacent to optic fiber cables. By measuring the introduced heating power and variation of temperature in the cable the seepage velocity can be estimated based on their method. Their study demonstrated that the detection of small flow velocity requires a higher heating power. Also, the higher heating power [67] and increased heating time [51] can highlight the seepage anomalies better.

The application of active DTS measurement in an embankment dam was tested by embedding optical cable horizontally into the downstream face of the embankment dam along its longitudinal axis [4]. The measurements proved the ability of the active system for detecting leakage as well as identifying the presence and location of the wet zone in embankment dams.

The active DTS technique can be used to determine the degree of saturation of the soil, where the seepage velocity has not taken place yet. In the absence of a seepage flow, the heat transfer is limited to the conduction in the soil. The effective thermal conductivity in the partly saturated soil depends on the solid particle thermal conductivity, the porosity, and the degree of saturation. The degree of saturation can be estimated [65] by measuring the temperature difference between the optical cable wall and the surrounding temperature after a heating period. An approximate solution is given also by Kristiansen [68] for the temperature difference between the cable wall and surrounding temperature, which is valid for long heating periods. Although the accuracy of the method for estimating the degree of saturation is not high, it is adequate to distinguish dry, moist, and saturated soil conditions [65]. 

An alternative method for measuring the water content was developed by Sayde et al. [66] based on the thermal response of the soil to the heat pulse in the form of the cumulative temperature increase over a certain period. Since the thermal conductivity and heat capacity increases with the water content, the cumulative temperature monotonically decreases with the water content. The method used this fact to calculate the water content from the temperature rise due to the heated optical fiber. More details on the method can be found in [66] and [69]. Recent works on the applications of active optical fiber DTS focus on the improvement of soil moisture determination by active DTS measurements [70,71,72].

Various researches on the active method show that the accuracy of seepage and the saturation estimation depends on the heating power and heating period [14,51,67]. For the detection of a concentrated leakage, a heat impulse of 3 to 5 Watts per meter of the cable length is enough, however, the measurement of the distributed flow velocity may require about 10 Watt per meter [65,73]. In addition to the heating power, the thermal response on the cable also depends on the cable cross-section. A smaller cross-section increases the span of the temperature difference in the zones with a small and fast seepage and leads to an easier evaluation of the seepage velocity in the soil [65].

The recent works on the DTS application include the development of the DTSGUI program, which is used for the processing of optical fiber DTS data [74]. DTSGUI is programmed in Python, which enables the users to edit, process, analysis, and visualize the obtained optical fiber DTS data. Such a program can be used to simplify the interpretation of temperature data for seepage detection.

The seepage detection methods presented in this section are compared in Table 2.

## 5. Discussion

The thermal analysis for the seepage monitoring relies on the accurate temperature measurement by the optical fiber DTS, proper installation of the optical fiber, and appropriate interpretation of temperature data to extract the seepage related information. The accurate temperature measurement is assured by the dynamic calibration of the measured data using internal and manual calibration techniques. The internal calibration requires assigning the differential power loss along the cable and can be supported by introducing reference sections.

The selection of the manual calibration technique and installation approach is based on the measurement requirements, on-site availabilities, and the prioritization of the measurements in terms of accuracy, time, and the area of investigation. The single-ended approach is appropriate for the long-distance measurements and has an easy calibration installation, but it neglects the local step power losses and, in the case of cable interruption, the measurement will not be possible beyond the cut location. Duplexed single-ended and double-ended measurements overcome these shortages but decrease the maximum length of measurement to a half. Besides, the accuracy of measurement at both ends of the cable decreases in the double-ended installation. Additionally, the duplexed cables generally include a fusion splice at the end of the cable, which may cause power losses that should be considered during the calibration [41]. The application of manual techniques for static calibration (where the calibration parameters are assumed constant over time) is a simple procedure. However, the manual techniques for the dynamic calibration and obtaining the calibration parameters for each measuring time step may require more effort and time-consuming procedures. McDaniel et al. [78,79] overcame this issue by performing the dynamic calibration developing a script-based technique and running it continuously on the obtained DTS data. The manual calibration techniques are compared in Table 1, Section 3 based on their potential advantages and disadvantages.

Seepage detection techniques are classified into two categories: active and passive. The application of the active method raises considerable concerns, especially in the case of monitoring at large structures. Heating a long optical fiber cable for monitoring large structures can be expensive or in some cases dangerous. In this method, the accuracy of measurements significantly depends on the heating power. ICOLD suggests that the length of the heated fiber should be shorter than two kilometers [2]. At the same time, any defects or breakdowns of the heating power source will influence the seepage monitoring system. With the recent advances in the optical fiber DTS and increasing temperature resolution, the passive measurement has been proven experimentally as a reliable method for seepage detection even in the presence of a low-temperature gradient between the seepage water and the soil [80].

Since the erosion by seepage may progress rapidly, it is crucial to detect seepage at the initiation phase. The selected interpretation method should be able to provide seepage information from the short-term measurements. The complexity of data processing in some of the interpretation techniques requires more time-consuming efforts. Another method that can be developed for the detection of seepage within the embankments from the temperature data is the machine learning (ML) technique. This technique has already been developed and used for the detection of seepage around the pipelines [81]. We also suggest that a coupled hydro-thermal numerical simulation should be performed for the intended structure. Such a simulation can be used for the proper installation approach and will provide beneficial information about the temperature distribution within the structure that can be used to interpret the obtained temperature data from the optical fiber DTS. Table 2 in Section 4 presents a comparative overview of the different seepage detection techniques which were reviewed in this paper.

## 6. Concluding Remarks

Various techniques for measurement data calibration and optical cable installation were reviewed in this paper. DTS systems provide dynamic internal calibration, however, sometimes manual calibration is required to obtain more precise temperature measurements. A comparative review of these methods was performed based on their accuracy, installation approaches, complexity, and the monitoring area. This comparison can be used for selecting the proper calibration technique of raw DTS data. The dynamic calibration for long-term measurements using the manual calibration requires more effort and time to apply the least-squares method to solve Equation (4) for each measuring time step. The development of an automatic fully dynamic calibration technique that can obtain the three calibration parameters for each measuring time step can be considered as a further topic of study.

In addition to the data calibration, we reviewed the passive and active DTS measurements and the techniques developed for temperature data interpretation. A comparison of these methods was presented. For early seepage detection, the selected method should extract the seepage related information from the short-term measurements with less possible complexity. An interpretation technique based on the comparison of the temperature measurements with simulated numerical results can be considered as a further study. The coupled hydro-thermal numerical simulation can be used to interpret the obtained temperature data from the optical fiber DTS.

## Figures and Tables

**Table 1:** Comparative overview of the manual calibration techniques.

Manual Calibration Methods			
Methods	Single-End Configuration		Double-End Configuration
References	[31,40,41]		[42,43,44,45]
	Simple Single-Ended	Duplexed Single-Ended	
**Potential** **advantages**	Less noise in the measurement process than the double-ended calibration technique. Easy calibration installation. Cover a bigger area for temperature monitoring.	Ability to correct non-uniform attenuation (step losses) along the cable. Reference sections located adjacent to the DTS instrument could be enough. Easy calibration installation.	Reference sections located adjacent to the DTS instrument are enough. The calibration can be achieved by only one reference with a stable absolute temperature. Capable to correct non-uniform attenuation. Ability to continue the temperature measurement from the other side in case of interrupted optical cable in the structure.
**Potential** **disadvantages**	Assuming a linear calculation of differential attenuation. Lack of capability for step power losses correction due to local effects. Requires a reference section close to the end of the cable. In the case of optical cable interruption, the measurement cannot continue beyond the defect location.	The maximum length of the measurement decreases to half.	Complex installation and set-up process. Generally nosier signals, especially near the DTS instrument. Requires very precise alignment of the cable. The maximum length of the measurement decreases to a half.

**Table 2:** Comparison of different techniques for DTS application in seepage detection.

Methods	Theory	Potential Advantages	Potential Disadvantages
**Lag-time** [7,52]	(Passive measurement) Comparison of temperature variation within the embankment with the seasonal temperature variation of air and water at the boundaries.	Simple process and easy interpretation Estimation of seepage flow velocity	One-dimensional assumption. Neglecting the heat conduction process.
**Source separation** [54,56,57,59]	(Passive measurement) Statistical and signal processing-based analysis to process the thermal data and to eliminate irrelevant factors from the leakage information.	Able to recognize the different sources that affect the thermal behaviour of the dam. Quantifying the influence of each source on the thermal behaviour.	Not able to estimate the flow velocity. Complex processing is required. The dependency of results on the parameters of the assigned sources.
**Singularities detection** [55,56]	(Passive measurement) The singularities (e.g., leakage and drains) present different trends of temperature variations compared to the non-singularity’s trends.	Can be used for alarm monitoring system and early leakage detection. The method is based on the relative changes in temperature and precise absolute temperature measurements are not a requirement.	The selection of the reference temperature vector is a challenge. Does not provide any information about the source of singularity in temperature vector.
**IRFTA** [9,61,63,75]	(Passive measurement) IRFTA employs Green’s function for the coupled water and heat transfer to show the thermal behaviour of the dam as an impulse response. function of the water and air temperature.	The significance of each air and water parameters in temperature variation can be estimated. The time lag both for water and air influences can be estimated. The parameters in this method may have a clear physical interpretation.	Relatively complex processing is required. The model describes only the linear behaviour of heat transfer.
**Active method** [10,14,51,65,73,76,77]	(Active measurement) Introducing heat into the embankment and monitoring the heat dissipation due to the presence of seepage or moisture.	The ability to estimate seepage velocity and degree of saturation. Applicable where the temperature gradient between the water and soil is small.	The length of cable for measurement is mostly limited to less than 2 km. For large structures, high heating power is required.
